# Reversible changes in the orientation of gold nanorod arrays on polymer brushes†

**DOI:** 10.1039/d0na00315h

**Published:** 2020-05-22

**Authors:** Yu Sekizawa, Hideyuki Mitomo, Mizuki Nihei, Satoshi Nakamura, Yusuke Yonamine, Akinori Kuzuya, Takehiko Wada, Kuniharu Ijiro

**Affiliations:** Graduate School of Life Sciences, Hokkaido University Kita 10, Nishi 8, Kita-Ku Sapporo 060-0810 Japan; Research Institute for Electronic Science, Hokkaido University Kita 21, Nishi 10, Kita-Ku Sapporo 001-0021 Japan mitomo@poly.es.hokudai.ac.jp ijiro@poly.es.hokudai.ac.jp; Global Institution for Collaborative Research and Education (GI-CoRE), Hokkaido University Kita 21, Nishi 10, Kita-Ku Sapporo 001-0021 Japan; Graduate School of Environmental Science, Hokkaido University Kita 10, Nishi 5, Kita-Ku Sapporo 060-0810 Japan; Graduate School of Chemical Sciences and Engineering, Hokkaido University Kita 13, Nishi 8, Kita-Ku Sapporo 060-8628 Japan; Department of Chemistry and Materials Engineering, Kansai University 3-3-35 Yamate, Suita Osaka 564-8680 Japan; Institute of Multidisciplinary Research for Advanced Materials, Tohoku University 2-1-1, Katahira, Aoba-ku Sendai 980-8577 Japan

## Abstract

Nanoparticles exhibit a number of unique properties such as localized surface plasmon resonance (LSPR). As this LSPR is sensitive to geometrical or spatial conditions, the arrangement of nanoparticles, in particular the active arrangement of plasmonic structures, is an important issue. In this study, gold nanorod (GNR) arrays were prepared by GNR attachment on anionic polymer (DNA) brushes *via* electrostatic interactions and their stimuli-responsive changes in orientation were investigated. As a result, the orientation of GNR arrays on DNA brushes reversibly changed by the modulation of electrostatic interactions between GNRs and polymers *via* changes in the solution pH. As these extensive GNR arrays are prepared *via* easy bottom-up processes, GNR surface properties are easily tuned by simple modification, and DNAs could be replaced with various synthetic polymers, we believe that this study will lead to the development of next-generation materials and devices with actively tunable structures.

## Abstract 2

Gold nanorod (GNR) arrays were prepared by GNR attachment on anionic polymer brushes *via* electrostatic interactions and their orientation reversibly changed by the modulation of interactions between GNRs and polymers *via* changes in the solution pH.

## Graphical abstract

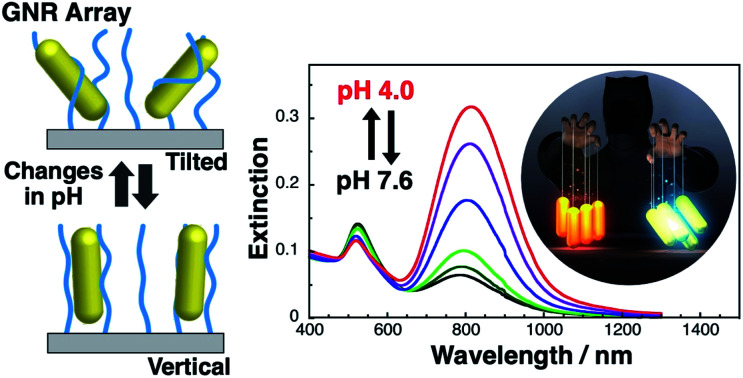

Nanoparticles exhibit a number of unique optical, magnetic, and electronic properties. One notable example of these properties is localized surface plasmon resonance (LSPR), which is induced by strong light-metal interactions, on metal nanoparticles. As LSPR is sensitive to geometrical or spatial conditions, the arrangement of metal nanoparticles is an important issue.^1–8^ Recently, the active arrangement (reversible tuning) of plasmonic structures has attracted a good deal of attention as a challenging new field.^9–13^ This arrangement is classified broadly into two approaches; gold nanoparticles (GNPs) are controlled as (i) dispersions in a solution or (ii) on/in substrates. A typical example of the former approach is stimuli-responsive assembly/disassembly using temperature-,^14–17^ pH-,^18–20^ and photo-responsive^21,22^ GNPs. For gold nanorods (GNRs), which exhibit two specific plasmonic absorptions for transverse and longitudinal localized surface plasmon resonance (T- and L-LSPR), factors related to their control become more complicated.^23^ As there are two types of ordering of the assembled structures, known as side-by-side and end-to-end assembly causing blue-shift and red-shift of L-LSPR, respectively, the assembly configuration must be properly controlled.^24–26^ The orientation of GNRs to the incident light is also a significant factor in L-LSPR excitation. Thus, the active control of GNR orientation when dispersed in solution was performed with the aid of a liquid crystal,^27^ and magnetic nanoparticles,^28^ and the application of an electric field.^29^

On the other hand, arrays fabricated on substrates provide an excellent platform for practical applications due to the various advantages in terms of their ease of handling, long-term stability, and so on. Thus, the latter approach, such as embedding into or attachment onto soft materials such as elastomers and gels as a supporting matrix, is promising as it allows their distances to be tuned through stretching or compressing the matrices by mechanical forces^30–32^ or the changing of the matrix volume through variations in the swelling conditions.^33–35^ These changes in the matrix shape were also applied to actively change the GNR orientations.^36^ From the viewpoint of applications, arrays on solid substrates are more preferable.^7,8^ Therefore, polymer brushes, which consist of a soft matrix attached on a solid substrate, are the best candidate. To date, a number of actively tunable plasmonic arrays based on changes in the nanoparticle distance on polymer brushes have been reported.^34,37–40^ However, there are no reports of the active control of GNR orientation using polymer brushes on solid substrates, despite their obvious importance.

In our previous study, we developed a preparation method for vertically aligned GNR arrays using polymer brushes as a template.^41,42^ As the polymer brushes are soft matrices, it is expected that the GNR orientation can be actively tuned through some kind of stimulation. In those reports, we found that GNRs formed vertically aligned arrays when mildly cationized GNRs, which were modified with 20% cationic and 80% nonionic ligands, were attached on negatively charged polymer (DNA) brushes *via* moderate electrostatic interactions. On the other hand, highly cationic GNRs, which were modified with 100% cationic ligands, showed a tilted orientation due to the conformational changes in the polymer brushes from too strong interactions between the GNRs and polymers. This finding suggests that modulation of the electrostatic interactions between GNRs and polymers can induce reversible changes in the GNR orientation between vertical and non-vertical on solid substrates. Although our previous reports showed the vertical alignment of GNRs as a static state, we did not achieve active changes in GNR orientation on polymer brush substrates, despite their great novelty and advantages. Thus, in this study, we prepared cationic GNRs coated with alkanethiol ligands, including 10% with the primary amino group at the terminus, and investigated their changes in orientation on the polymer brushes composed of double- and single-stranded DNAs as rigid and flexible anionic polymers, respectively, *via* changes in their surface potential through variations in solution pH, which is one of the widely used factors for changing chemical and/or physical properties (Scheme 1).

**Scheme 1:**
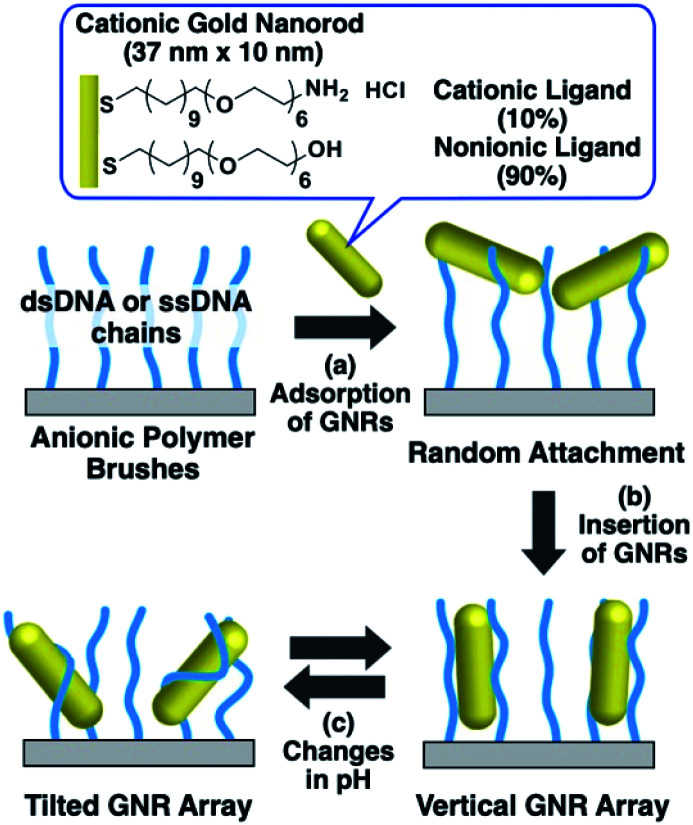
Preparation of vertical gold nanorod arrays and their pH-responsive changes in orientation.

First, we prepared vertically aligned GNR arrays using double-stranded DNA (dsDNA) brushes as a template according to our previous paper.^42^ Briefly, after streptavidin immobilization on the quartz surfaces, biotinylated dsDNA, which has a 148-bp random sequence, was immobilized through avidin-biotin interactions, providing dsDNA brush substrates. The DNA density was calculated to be *ca.* 21 000 ± 3300 chains per μm^2^ (7.5 ± 0.5 nm as an average DNA interchain distance) (Fig. S1†). Then, 10% cationic GNRs (37 × 10 nm) were attached on the dsDNA brushes *via* electrostatic interactions in Milli-Q water (Scheme 1a, Fig. S2†). The reason why we used 10% cationic GNRs in this study is that highly cationic GNRs are expected to show smaller orientation changes from the originally tilted state to the more tilted state and also stronger interactions between GNRs and DNAs prevent structural changes through the rearrangement of ionic bonding. Unadsorbed GNRs in the bulk solution were removed by replacement of Milli-Q water 3 times. When the solution was changed with the buffer (10 mM Tris–HCl, pH 7.6), the L-LSPR peak at around 800 nm gradually decreased for over several hours without any significant changes in the extinction of T-LSPR around 520 nm (Fig. 1a and S3†). This decrease in the L-LSPR-specific peak indicates a change in the alignment of GNRs from random to vertical as it originates from the angle dependence of incident light as mentioned above (Scheme 1b). The extinction spectra under polarized light strongly support the vertical alignment of GNRs in the buffer solution, the same as our previous report (Fig. 1b).^41^ A p-polarized light-specific L-LSPR peak can be observed around 640 nm (highlighted in yellow). This blue-shift from the original L-LSPR peak at around 800 nm indicates plasmon coupling in a side-by-side configuration, which also supports vertical array formation. Based on our previous simulation results, we estimate the center-to-center distance of the 10 nm GNRs to be *ca.* 30 nm (a gap distance of *ca.* 20 nm).^41^ Further, we evaluated the GNR density on this array to be *ca.* 1500 particles per μm^2^ on 21 000 chains per μm^2^ dsDNA brushes by a comparison of the extinction value in Fig. 1a with that in our previous report as the extinction value at 400 nm is a simple indicator of the number of GNRs due to their absorption and scattering.^42^ In addition, the vertically aligned arrays after incubation in buffer (pH 7.6) did not show any spectral change on replacement of the solution back to Milli-Q water (Fig. S4†). These results indicated the successful preparation of vertically aligned GNR arrays with enough free spaces around each GNR to afford changes for an energetically stable configuration (Scheme 1b).

**Fig. 1:**
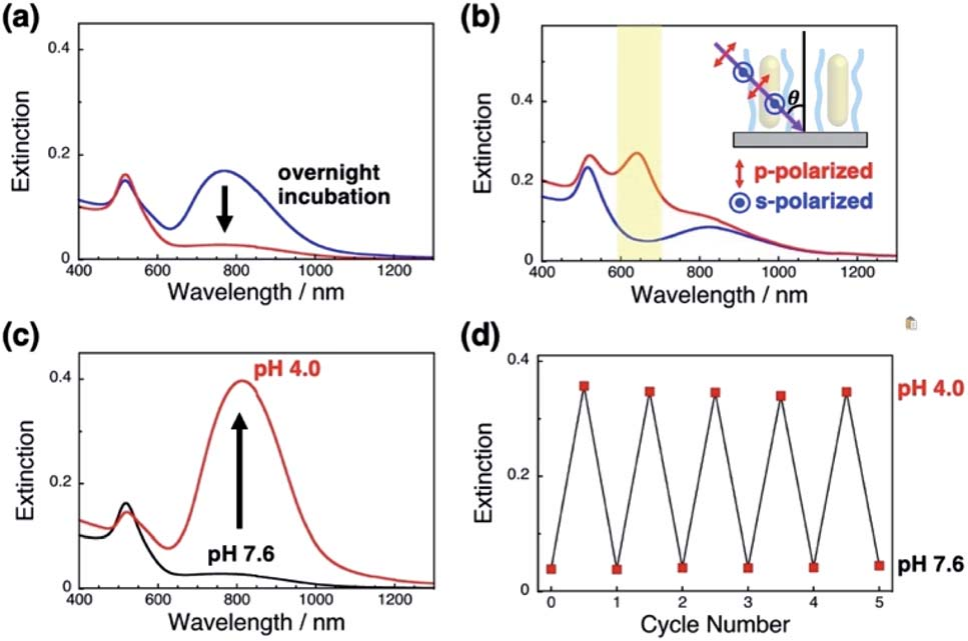
Extinction spectra of GNRs attached on dsDNA brushes. (a) Time-dependent spectral change after replacement of the solution with the buffer; soon after replacement (blue) and after overnight incubation (red). (b) Extinction spectra after overnight incubation measured under p-polarized (red) and s-polarized light (blue) at *θ* = 45°, where *θ* is the angle of the incident light to the substrate. (c) Extinction spectra for a change in pH from 7.6 (black) to 4.0 (red). (d) Extinction values for the L-LSPR peak for repeated changes of pH between 7.6 and 4.0.

Next, we examined their configuration changes by the replacement of buffer solutions. The zeta-potentials of the cationic GNRs gradually increased from +1 to +15 mV as the pH decreased from 10 to 4.0 (Fig. S5a†). This gradual increase is thought to be a nearest neighbor effect of the amino groups on a self-assembled monolayer.^43,44^ On the other hand, as expected, the dsDNA did not show any significant changes in zeta-potential between pH 7.6 and 4.0 (−34 to −39 mV) (Fig. S5b†). Thus, we changed the pH in this range. When pH fell to pH 4.0, the L-LSPR peak at around 800 nm increased tremendously together with a slight decrease in the T-LSPR peak at around 520 nm (Fig. 1c). As explained above, this increase in L-LSPR intensity indicates that the vertical alignment of the GNRs changed to a tilted alignment due to the change in pH.^41^ As this change is thought to include plasmon coupling effects, it is difficult to estimate the tilt angles. However, this is quite a large change compared to those in the previous papers on dispersed GNR orientation tuning by magnetic or electric fields, even though the GNRs were immobilized on polymer brushes in our system.^28,29^ Importantly, this pH-responsive change in intensity (orientation) is very quick (about a second) and reversible for at least several cycles (Scheme 1c, Fig. 1d, Movie S1, and Fig. S6†). Although we could not obtain direct images such as SEM or AFM images yet due to technical difficulties, these plasmonic spectra from GNR unique characteristics strongly support reversible changes in GNR orientations.

A more detailed investigation on pH-responsiveness was performed (Fig. 2). When pH was decreased stepwise from 7.6 to 4.0, the L-LSPR intensity increased, especially between pH 4.5 and 4.0 (Fig. 2a). Also, when the pH was increased from 4.0 to 7.6, the L-LSPR intensity decreased, especially between 4.5 and 5.5, and returned to the original intensity at pH 6.0 (Fig. 2b). The L-LSPR intensities with regard to change in pH are plotted in Fig. 2c, which shows that the L-LSPR intensity changed markedly between pH 4.0 and 5.5. It is noteworthy that there is a clear hysteresis. As the pH decreases, amino groups at the GNR surface are protonated and form ionic bonds with the dsDNA. As the system gains a large energetic merit from this ionic bond formation, it becomes energetically more stable. When this energy gain is larger than the energy loss from DNA bending or disarrangement, the DNA configuration is thought to change, causing a change in GNR orientation. This kind of energetic relationship is supported by theoretical reports.^45^ On the other hand, as the pH increases, amino groups at the GNR surface are deprotonated and their ionic bonding is disrupted, causing changes in both the dsDNA and GNR configuration *via* energy loss and gain. Thus, it is expected that these energy gain–loss relationships, which represent energetically different pathways, can explain the observed hysteresis.^46^ In other words, this hysteresis supports our notion that the GNR orientation can be tuned by changes in the interactions between the anionic polymers and moderately cationic coated GNRs on the dsDNA brushes.

**Fig. 2:**
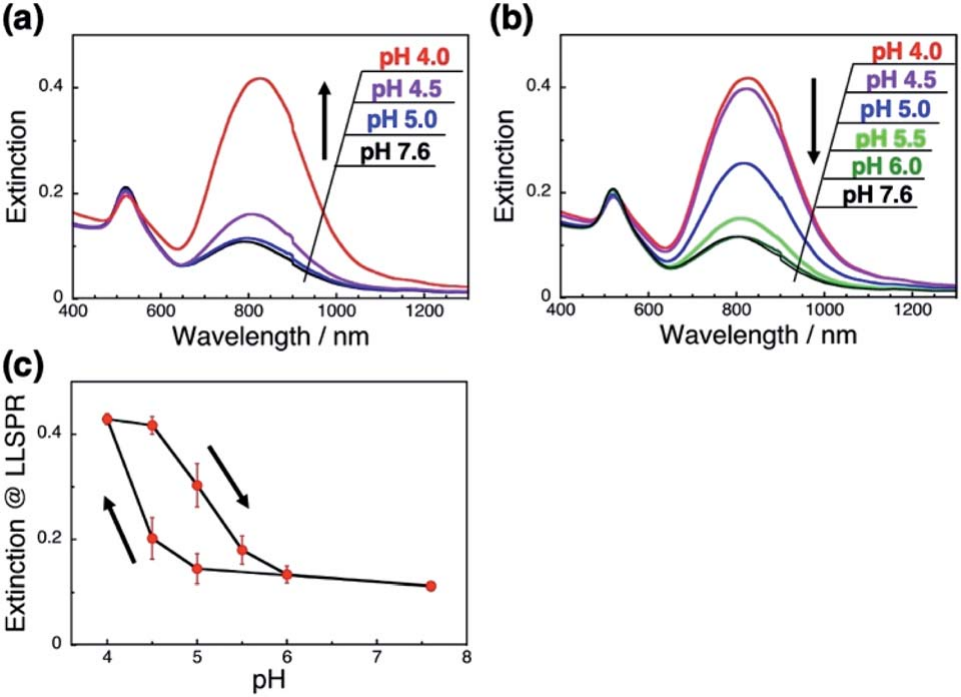
pH-Responsive spectral changes for the GNRs on dsDNA brushes. (a) A decrease in pH from 7.6 to 4.0. (b) An increase in pH from 4.0 to 7.6. Spectra are shown in black (pH 7.6), green (pH 6.0), light green (pH 5.5), blue (pH 5.0), purple (pH 4.5), and red (pH 4.0). (c) Extinction values for the L-LSPR peak according to pH. Error bars represent standard deviation of 3 repeated cycles.

To clarify the mechanism underlying this pH-responsive change in GNR orientation on dsDNA brushes between pH 4.0 and 5.0, which suggests another dominant factor other than the electrostatic interactions between DNA and GNRs, we checked the effects of pH on the LSPR of the GNRs and the absorption of the dsDNA by spectroscopy. Cationic GNRs did not show any changes in extinction spectra (Fig. S7†). On the other hand, the dsDNA on the substrates showed a spectral change in this pH range (Fig. 3a and b). When the pH fell to 4.0, the absorption peak red shifted from 258 nm to 265 nm and *A*_260_/*A*_280_ also showed a change from *ca.* 1.8, which is a normal value for dsDNA, to 1.2. It is noticeable that this change was marked between pH 4.0 and 5.0. Further, this spectral change was also reversible (Fig. S8†). To obtain more information about the structure of the dsDNA on the substrates, we examined the effects of pH on the circular dichroism (CD) spectra (Fig. 3c). The CD spectra of the dsDNA brushes showed a negative band at around 250 nm and a positive band at around 280 nm under neutral pH conditions. The data indicated that the dsDNA brushes prefer a B-type double-stranded structure on the substrates in neutral buffer solutions (pH 7.6 to 5.0).^47^ In sharp contrast, a drastic change in the dsDNA structure was induced by decreasing the pH down to 4.0 from 5.0 as indicated by the CD spectral change. These spectral studies of absorption and CD show that the orientation of the GNR arrays on the dsDNA brushes was reversible through changes in the solution pH accompanied with DNA conformational changes, suggesting that pH-dependent conformational changes in dsDNA, rather than electrostatic interactions, are a dominant factor contributing to pH-responsive changes in GNR orientation on dsDNA brushes. It is expected that dsDNA is too rigid to bend or be rearranged under neutral pH conditions due to its double helical structures; however, when the DNA helix structure is deformed by a decrease in pH (<pH 5), it becomes more flexible and allows changes in GNR orientation *via* change in the interactions between the DNA and GNRs.

**Fig. 3:**
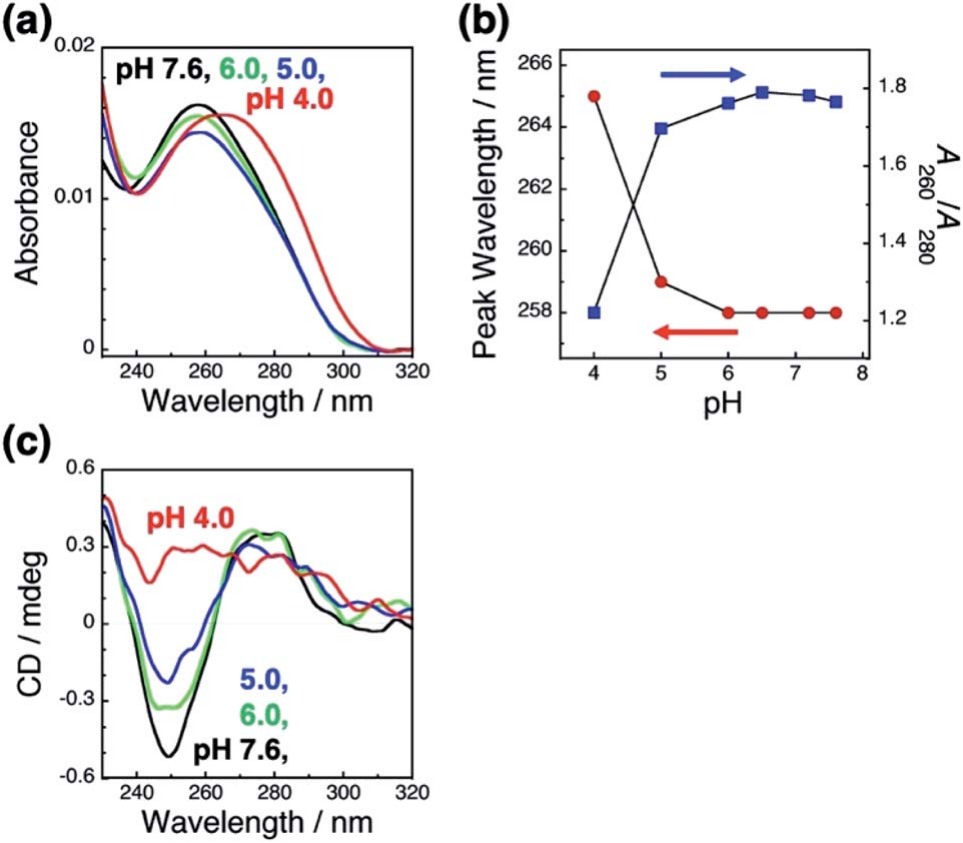
Absorption spectra of dsDNA brushes in the buffer at various pH values (a) and the peak wavelength (blue) and absorbance ratio of *A*_260_/*A*_280_ (red) at each pH value (b). (c) Circular dichroism (CD) spectra of dsDNA brushes in the buffer at various pH values. Absorption and CD spectrum at each pH value are shown in black (pH 7.6), light green (pH 6.0), blue (pH 5.0), and red (pH 4.0).

As it is expected that the rigidity of the polymer is an important factor for the change in the orientation of GNRs on polymer brushes, we next applied a flexible polymer to this system. Fortunately, we have already reported that the vertical GNR alignment can also be performed on single-stranded DNA (ssDNA) brushes,^42^ which are more flexible and share the same lack of well-ordered structures as general synthetic polymers.^48,49^ Thus, we investigated the tuning of GNR orientation by changes in the interactions between polymers and GNRs on ssDNA brushes. To avoid any effects of pH on the polymer itself, we used 148-base poly(dT) as the ssDNA. We successfully prepared the ssDNA brushes of *ca.* 19 000 ± 880 chains per μm^2^ (Fig. S9a†) and vertical GNR arrays (Fig. S10†) by the same procedures as described above. As expected, ssDNA[poly(dT)] did not show any significant changes (shift) in absorption spectra or the zeta-potential when the pH was changed between 7.6 and 4.0 (Fig. S9bc†). When the pH was decreased from 7.6 to 4.0, the L-LSPR intensity gradually increased, suggesting a gradual change in GNR orientation (Fig. 4a). Also, when the pH was increased from 4.0 to 7.6, the L-LSPR intensity decreased and returned to the original value (Fig. 4b). The L-LSPR intensities with regard to the changes in pH are plotted in Fig. 4c, which shows that L-LSPR intensity changed between pH 4.0 and 7.6. This spectral change is gentler and in a wider pH range than that observed for dsDNA. This is well correlated with the changes in the zeta-potential according to pH (Fig. S5a†). A clear hysteresis was also observed as with the dsDNA brushes. These results perfectly met our expectation, and indicate that the reversible change in the orientation of the GNR arrays on polymer brushes was induced by the modulation of the electrostatic interactions between the GNRs and polymers *via* changes in the solution pH.^46^ As ssDNA possesses a similar persistence length to synthetic polymers, these findings indicate that a broad range of synthetic stimuli-responsive polymers could be applicable to this approach, leading to a wide range of applications.

**Fig. 4:**
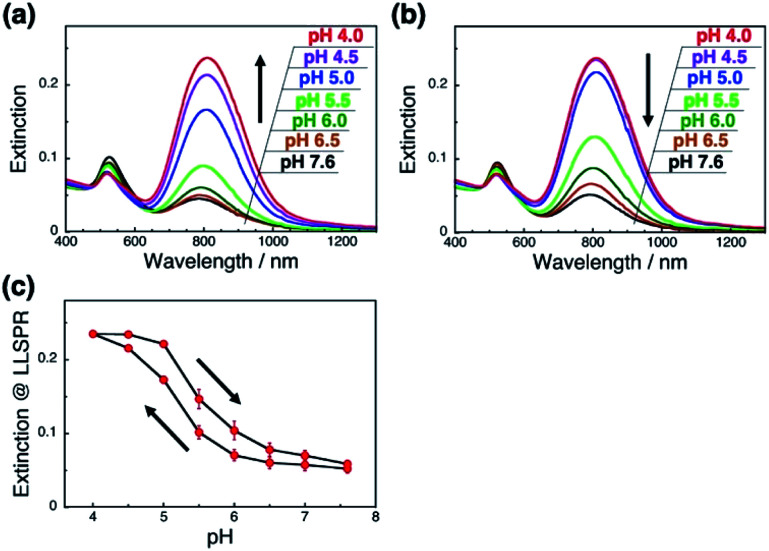
pH-Responsive spectral changes for GNRs on ssDNA[poly(dT)] brushes. (a) An increase in pH 7.6 to 4. (b) A decrease in pH from 4.0 to 7.6. Spectra are shown in black (pH 7.6), brown (pH 6.5), green (pH 6.0), light green (pH 5.5), blue (pH 5.0), purple (pH 4.5), and red (pH 4.0). (c) Extinction values for the L-LSPR peak with regard to pH changes. Error bars represent standard deviation of 3 repeated cycles.

In conclusion, we prepared GNR arrays attached on anionic polymer (ds and ssDNA) brushes *via* electrostatic interactions and investigated their pH-responsive changes in orientation. As the GNRs were modified with 10% amine-terminated ligands, the zeta-potential of the GNRs gradually increased with decrease in the solution pH. The GNR orientation on the DNA brushes was reversibly changed by the modulation of the electrostatic interactions between the GNRs and polymers *via* changes in the solution pH. The L-LSPR peak change is gentler and in a wider pH range (pH 4.0–7.6) on ssDNA brushes, while the L-LSPR peak changed drastically and in a narrow pH range (pH 4.0–5.0) on the dsDNA brushes. We consider that dsDNA is too rigid to bend or be rearranged at a neutral pH due to its double helical structure. However, when the DNA helix structure is deformed by a decrease in pH (<pH 5), it will behave like a soft polymer and then GNR orientation can be tuned *via* changes in the interactions between the DNA and GNRs. As these extensive GNR arrays are prepared *via* easy bottom-up processes, GNR surface properties are easily tuned by simple modification, and DNAs could be replaced with various synthetic polymers, we believe that this study will lead to the development of next-generation materials and devices with actively tunable structures.

## Conflicts of interest

There are no conflicts to declare.

## Supplementary Material

NA-002-D0NA00315H-s001

NA-002-D0NA00315H-s002
