# Supplementary material for: Reversible changes in the orientation of gold nanorod arrays on polymer brushes
Source: Nanoscale Adv. 2020 May 22;2(9):3798–803. doi: 10.1039/d0na00315h (PMC9418524; doi:10.1039/d0na00315h)
Supplement: NA-002-D0NA00315H-s001 [file NA-002-D0NA00315H-s001.pdf]

Electronic Supplementary Material (ESI) for Nanoscale Advances.
This journal is © The Royal Society of Chemistry 2020

Reversible Changes in the Orientation of Gold Nanorod Arrays on Polymer Brushes

Yu Sekizawa [a] *Hideyuki Mitomo,* [*bc] *Mizuki Nihei,* [d] *Satoshi Nakamura,* [e†] *Yusuke Yonamine,* [bc]

Akinori Kuzuya, [f] *Takehiko Wada,* [g] *and Kuniharu Ijiro* [*bc]

[a] Graduate School of Life Sciences, Hokkaido University, Kita 10, Nishi 8, Kita-Ku, Sapporo 060-0810, Japan

[b] Research Institute for Electronic Science, Hokkaido University, Kita 21, Nishi 10, Kita-Ku, Sapporo 001-0021, Japan

[c] Global Station for Soft Matter, Global Institution for Collaborative Research and Education, Hokkaido University, Kita 21, Nishi 11, Kita-Ku, Sapporo 001-0021, Japan

[d] Graduate School of Environmental Science, Hokkaido University, Kita 10, Nishi 5, Kita-Ku, Sapporo 060-0810, Japan

[e] Graduate School of Chemical Sciences and Engineering, Hokkaido University, Kita 13, Nishi 8, Kita-Ku, Sapporo 060-8628, Japan

[f] Department of Chemistry and Materials Engineering, Kansai University, Osaka 564-8680, Japan

[g] Institute of Multidisciplinary Research for Advanced Materials, Tohoku University, 2-1-1, Katahira, Aoba-ku, Sendai 980-8577, Japan

[†] Present address: National Institute of Advanced Industrial Science and Technology (AIST),

2266-98 Anagahora, Shimo-Shidami, Moriyama, Nagoya 463-8560, Japan.

Experimental

Extinction spectra were recorded with a V-770 UV-vis spectrometer (JASCO Corp., Japan). In the extinction spectrum analysis, unless otherwise stated, the incident light was non-polarized and its direction was perpendicular to the plane containing the adsorbed GNRs. Fluorescence intensity measurements were performed with a Pharos FX Molecular Imager (Bio-Rad Laboratories Inc., USA). Scanning transmission electron microscopic (STEM) images were obtained using a STEM HD-2000 system (Hitachi High-Tech Manufacturing & Service Co., Ltd., Japan) with a 200 kV acceleration voltage. The zeta-potentials of cationic GNRs and DNAs were measured with a Zetasizer Nano ZS (Malvern Panalytical Ltd. UK) using disposable folded capillary cells. The circular dichroism (CD) spectra of dsDNA brushes were measured with a J-820 system (JASCO Corp., Japan) with 10-times integration.

The buffers used in this study were as followings; 10 mM acetate buffer (pH4.0, 4.5, 5.0, and 5.5), 10 mM 2-(N-morpholino)ethanesulfonic acid (MES) buffer (pH 6.0 and 6.5), and 10 mM Tris(hydroxymethyl)aminomethane (Tris-HCl) buffer (pH 7.0 and 7.6). Buffer replacement was repeated 3 times for the complete change in solution pH.

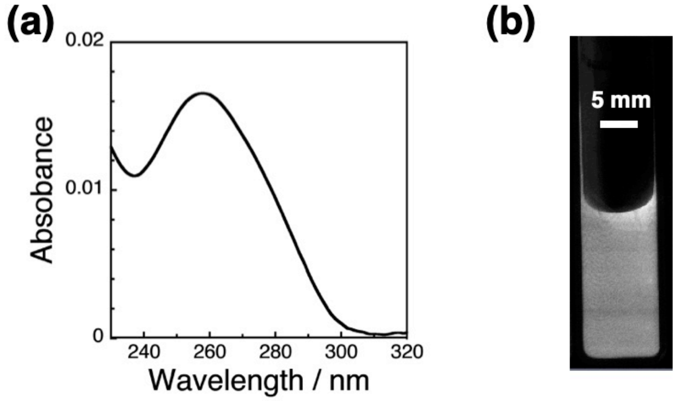

Figure S1. Absorption spectrum (a) and fluorescent image (b) of the dsDNA brushes. The DNA density was calculated as ca. 21000 ± 2900 chains/μm^2 (7.5 ± 0.5 nm as an average DNA interchain distance) from the absorbance at 260 nm and ca. 25,000 ± 1900 chains/μm^2 (6.8 ± 0.3 nm as an average DNA interchain distance) from the fluorescence intensity of Alexa Fluor 647 at the terminus, respectively, according to our previous report.[1]

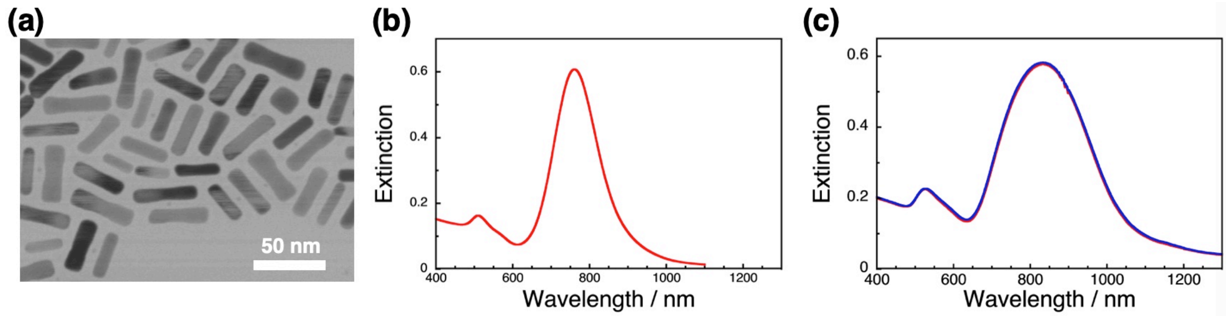

Figure S2. STEM image (a) and extinction spectrum (b) of prepared GNRs. (c) Extinction spectrum of adsorbed GNRs on dsDNA brushes in Milli-Q water. GNR size was determined as 9.9 ± 2.0 nm in diameter and 37 ± 4.7 nm in length (300 particles counted). Extinction spectra did not show any changes after incubation for 12h (blue) in Milli-Q water, suggesting a kinetically stable GNR configuration in a random orientation. Broadening of this L-LSPR peak was expected to derive from plasmon coupling effects on a random distribution.

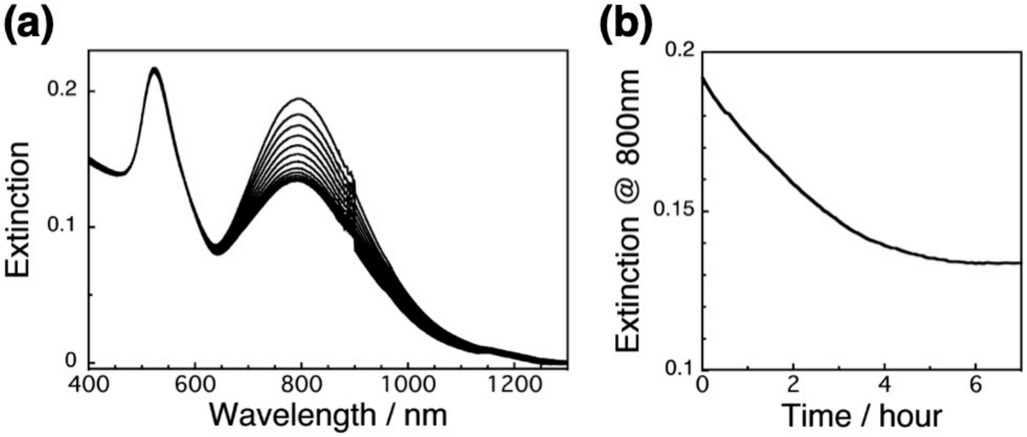

Figure S3. (a) Extinction spectra of GNRs attached on the dsDNA brush after replacement of the solution to the buffer and (b) time-dependent changes in L-LSPR intensity.

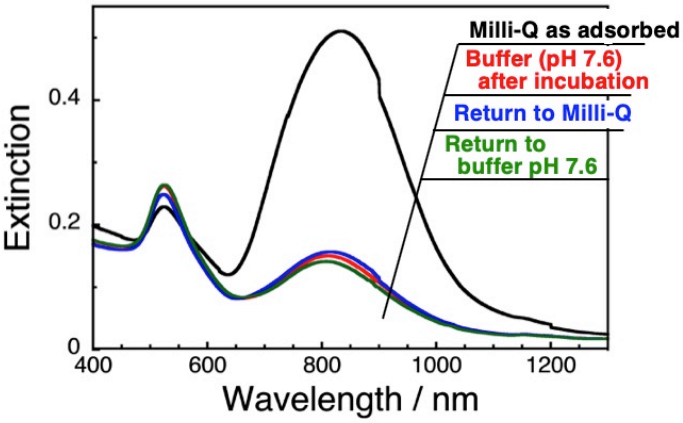

Figure S4. Extinction spectra of GNRs attached on the dsDNA brushes as adsorbed in Milli-Q water (black), after overnight incubation in 10 mM Tris-HCl buffer, pH 7.6, (red), return to Milli-Q water after incubation in the buffer (blue), and return to buffer again (green).

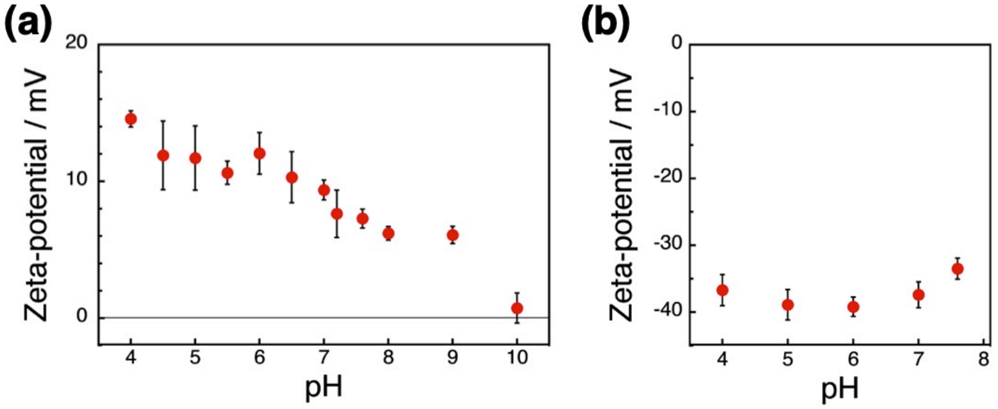

Figure S5. Zeta-potential of 10% cationic ligand-modified GNRs (a) and 148bp-dsDNA (b) in a buffer solution.

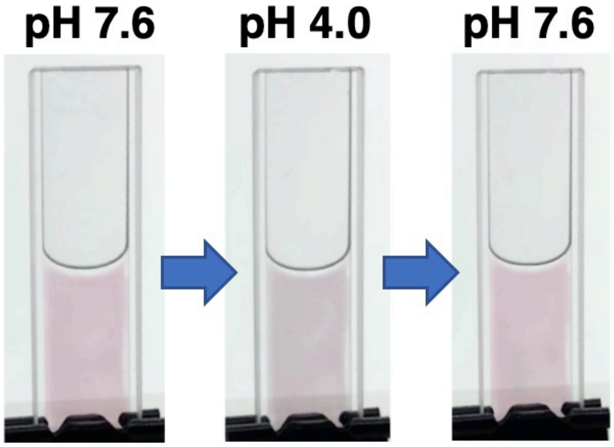

Figure S6. Snapshot of pH-responsive color changes for the the GNRs attached on the dsDNA brushes. These are taken from the Movie S1. The color change is not drastic because the large spectral changes are observed in Near-IR region.

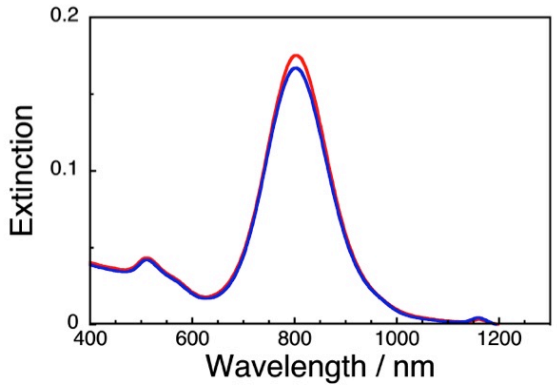

Figure S7. Extinction spectra of 10% cationic GNRs at pH 7.6 (blue) and 4.0 (red).

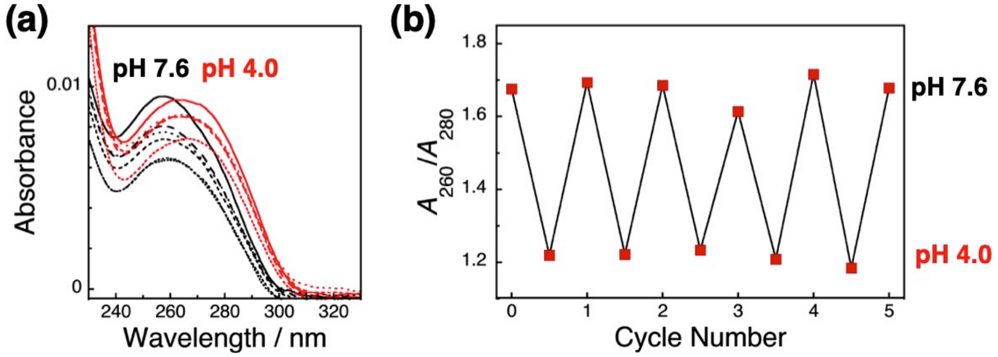

Figure S8. Absorption spectra of dsDNA brushes (a) and absorption ratio as A_{260}/A_{280} (b) on repeated changes in buffer pH from 7.6 to 4.0. Absorption spectra are shown as black (pH 7.6) and red (pH 4.0).

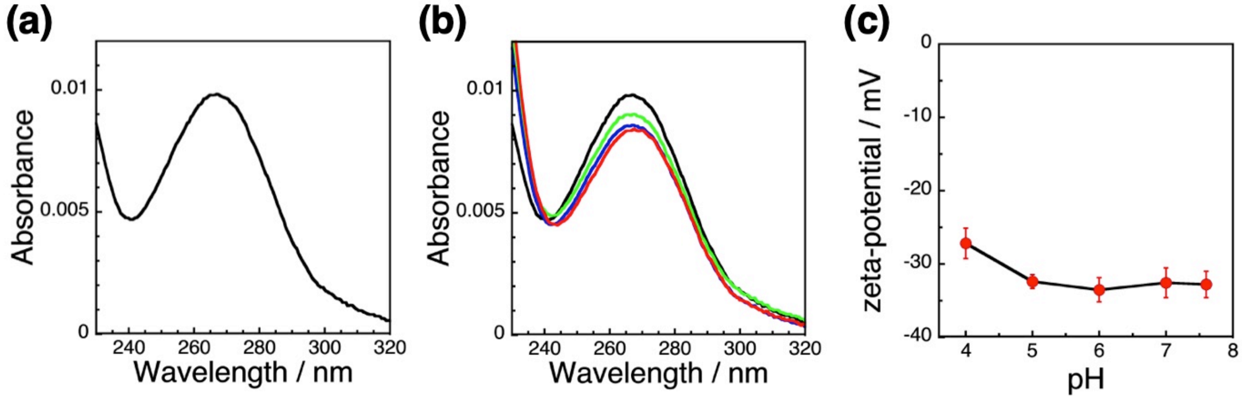

Figure S9. (a) Absorption spectrum of the prepared ssDNA [poly(dT)] brushes. The DNA density was calculated as ca. 19000 ± 880 chains/μm^2 (7.9 ± 0.2 nm as an average DNA interchain distance) from the absorbance at 260 nm. (b) pH-Independence of ssDNA [poly(dT)] brushes on absorption spectra. Spectra are shown as black (pH 7.6), light green (pH 6.0), blue (pH 5.0), and red (pH 4.0). (c) Zeta-potential of ssDNA [poly(dT)] in solution at various pH values.

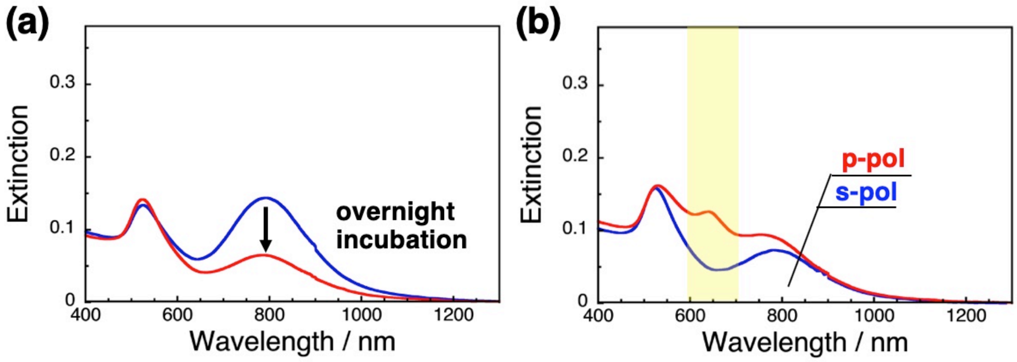

Figure S10. Extinction spectra of GNRs adsorbed on the ssDNA [poly(dT)] brushes. (a) Time-dependent spectral changes after replacement of solution to buffer; soon after replacement (blue) and after overnight incubation (red). (b) Extinction spectra after overnight incubation measured under p-polarized (red) and s-polarized light (blue) at $\theta = 45°$.

Reference

[1] S. Nakamura, H. Mitomo, Y. Sekizawa, T. Higuchi, Y. Matsuo, H. Jinnai, and K. Ijiro, *Langmuir*, 2020, **36**, 3990-3999.
